# UbcH10 a Major Actor in Cancerogenesis and a Potential Tool for Diagnosis and Therapy

**DOI:** 10.3390/ijms21062041

**Published:** 2020-03-17

**Authors:** Ivan Presta, Fabiana Novellino, Annalidia Donato, Domenico La Torre, Caterina Palleria, Emilio Russo, Natalia Malara, Giuseppe Donato

**Affiliations:** 1 Department of Health Sciences, University “Magna Græcia” of Catanzaro, 88100 Catanzaro, Italy; palleria@unicz.it (C.P.); erusso@unicz.it (E.R.); gdonato@unicz.it (G.D.); 2 Neuroimaging Unit, Institute of Bioimaging and Molecular Physiology, National Research Council (IBFM-CNR) Viale Europa, 88100 Catanzaro, Italy; fabiana.novellino@cnr.it; 3 Department of Medical and Surgical Sciences, University “Magna Graecia” of Catanzaro, 88100 Catanzaro, Italy; annalidia.donato@gmail.com (A.D.); dlatorre@unicz.it (D.L.T.); 4 Department of Clinical and Experimental Medicine, University “Magna Graecia” of Catanzaro, 88100 Catanzaro, Italy; nataliamalara@unicz.it

**Keywords:** cancer, ubiquitin proteasome system, UbcH10, cell cycle control, therapy, gene silencing, immunohistochemistry, prognosis

## Abstract

Malignant transformation is a multistep process in which several molecular entities become dysregulated and result in dysfunction in the regulation of cell proliferation. In past years, scientists have gradually dissected the pathways involved in the regulation of the cell cycle. The mitotic ubiquitin-conjugating enzymes UbcH10, has been extensively studied since its cloning and characterization and it has been identified as a constantly overexpressed factor in many types of cancer. In this paper, we have reviewed the literature about UbcH10 in human cancer, pointing out the association between its overexpression and exacerbation of cancer phenotype. Moreover, many recalled studied demonstrated how immunohistochemistry or RT-PCR analysis can distinguish normal tissues and benign lesions from malignant neoplasms. In other experimental studies, many of the consequences of UbcH10 overexpression, such as increased proliferation, metastasizing, cancer progression and resistance to anticancer drugs are reversed through gene silencing techniques. In recent years, many authors have defined UbcH10 evaluation in cancer patients as a useful tool for diagnosis and therapy. This opinion is shared by the authors who advertise how it would be useful to start using in clinical practice the notions acquired about this important moleculein the carcinogenesis of many human malignancies.

## 1. Introduction

Ubiquitination-dependent proteolysis is related to various cellular processes including cell cycle progression, signal transduction, and differentiation. Ubiquitin (Ub) is a small regulatory protein of 76 amino acids that is attached to other proteins as a post-translational regulatory event. The ubiquitylation of proteins that must be removed is carried out by a sequential cascade that involves three classes of enzymes: the ubiquitin-activating enzymes (E1s) create a thioester bond between a cysteine residue into the active site and the carboxyl end of ubiquitin, and then transfer it to another cysteine on ubiquitin-conjugating enzymes (E2s). The E2 enzymes, in turn, transfer the ubiquitin units to specific protein substrates, autonomously or engaging specific ubiquitin ligases (E3s) enzymes that probably help E2s to ensure the specificity of the substrate [1,2,3]. In the human genome, two genes coding for E1s, at least 38 genes encoding for E2s and about six hundred genes coding for E3s have been identified [4]. Modified substrate proteins are recognized and subsequently degraded by the 26S proteasome, a multisubunit ATP-dependent protease complex, consisting of a cylindrical shaped 20S core, capped on one or both sides by a 19S regulatory particle [5,6]. The entire system is named ubiquitin proteasome system (UPS). Some components among these ubiquitin tagging enzymes arrays have become specialized to perform their function within the cell cycle regulation. The anaphase-promoting complex/cyclosome (APC/C) is a multisubunit complex being the main core an E3 ligase. By interacting with E2~Ub (intermediates, it catalyses polyubiquitination of key cell cycle regulators, inducing the metaphase/anaphase transition exit from mitosis, and maintenance of G1 [7]). It has been shown that in eukaryotes cells a specific ubiquitin-conjugating enzymes (E2) (UbcH10 in humans, coded by *UBE2C* gene) has a crucial role into the timer properties of APC/C. Moreover, in the early 2000s, it was observed that UbcH10 is highly expressed in numerous cancerous cell lines and various primary tumors in comparison with surrounding normal tissues [8]. Since then, many studies have examined the expression of UbcH10 in many human tumors, highlighting its contribution in the mechanisms of carcinogenesis and assuming its usefulness in a diagnostic and therapeutic context. The aim of this work is to evaluate the state of the art of the knowledge about the impact of UbcH10 (over-)expression in cancer, and speculate on new diagnostic and therapeutic possibilities.

## 2. The Role in Cell Cycle Regulation

The run through the cell cycle is mainly based on the ordered and sequential activation of cyclin-depended kinases (CDKs) that, upon association with their partner cyclins, initiate crucial events and drive the transcription of genes important for cell cycle promotion. The timed activation of cyclin/CDK complexes is then mechanistically due to cyclins fluctuations, some of which are inducible by external stimuli (D-type cyclins), and others respond to internal mechanisms of induction (A-type and B-type cyclins), but all are promptly destroyed by ubiquitination and proteasomal degradation [9]. During the various phases of the cell cycle, several cyclins prevail to push the progression of a given phase or process and temporarily block the subsequent ones. Cyclin A is expressed from late G1 until prometaphase and its association with Cdk1 or Cdk2 leads to the inactivation of APC/C by inhibiting Cdh1 coactivator at the beginning of the S-phase [10,11]. Cyclin Ais, in turn, a substrate of APC/C that promotes its ubiquitinoylation and degradation from late G2. Subsequently, APC/C in its complete activation, promotes the degradation of securin, geminin, and cyclin B1, inducing sister chromatid separation and also promoting exit from mitosis [12,13,14]. In 1997, Townsley and co-workers cloned the human full-length complementary DNA (cDNA) for the *UBE2C* gene, the human homolog of clam E2-C type ubiquitin carrier protein and called it UbcH10 [15]. By using a dominant-negative mutant they demonstrated that the encoded protein is involved in the ubiquitin mediated degradation of cyclin A and cyclin B and then in the progression through mitosis [15].

Yamanaka and co-workers observed the ability of APC/C to regulate E2-C expression and proved in a mouse cell line, that mE2-C is able to polyubiquitylate itself with the involvement of the cys114 residue, into the active site [16]. This function, together with other mechanisms, as well as inhibition of the Cdh1 coactivator or expression of Emi1, are essential during G1 phase for switching the active APC/C into a cyclin A permissive state. Rape and Kirschner showed in human cell lines that cyclin A is stabilized in late G1, when instead the others APC/C substrates continue to be efficiently destroyed. This selectivity of substrates occurs only when the APC/C-specific ubiquitin conjugating enzyme (E2) UbcH10 is degraded in G1 until the prometaphase. UbcH10 in this way is eliminated at the M-G1 transition through ubiquitin mediated proteolysis [17].

## 3. UbcH10 Studies in Various Types of Tumors

Studies focusing on the role of UbcH10 in cancer have been conducted on human tissues by using immunohistochemical techniques, or in tumor-derived cell lines evaluating the *UBE2C* mRNA expression. Table 1 collects data from general literature.

### 3.1. Brain Tumors

Regarding brain tumors, there are studies on both meningiomas and gliomas. In meningiomas, high levels of UbcH10 appear to be associated with progression towards higher grades of malignancy and recurrence. The retinoblastoma protein-interacting zinc finger protein1 (RIZ1) is known to be an important tumor suppressor gene and negatively correlates with the pathological grade of meningioma and UbcH10 expression. It has been found in meningioma cells that RIZ1 is able to downregulate UbcH10 in a c-Myc-dependent manner and that provoked inhibitory on motility, invasion ability, and significantly increased the number of apoptotic cells [18,19]. The UbcH10 expression in astrocytic tumors is directly related to the tumor grade and no expression has been observed in normal tissue and gliosis. The expression levels of UbcH10 mRNA have been found elevated in high-grade versus low-grade astrocytomas or normal controls [20,21]. Furthermore, it has been shown that RNA interference (RNAi) targeting UbcH10 induced growth inhibition, apoptosis, and cell cycle arrest in U-251 glioblastoma cell line, proving the important role for UbcH10 in the regulation of proliferation, apoptosis, and cell cycle progression of glioma cells [22].

### 3.2. Lung Cancer

UbcH10 expression has been mainly explored in non-small cell lung carcinomas (NSCLC). In samples from poorly differentiated NSCLC, UbcH10 was overexpressed compared to well-differentiated ones. In particular, the expression levels were significantly higher in squamous cell carcinoma than in adenocarcinoma. It has been found that higher UbcH10 expression is associated with a shorter postoperative survival in NSCLC patients. Moreover, UbcH10 expression directly correlates with the mutational status of p53 while the correlation is inverse with that of EGFR [24,25]. Interestingly, in the lung, squamous cell carcinoma-derived SK-MES-1 cells line the suppression of UbcH10 inhibited cell proliferation, increased chemosensitivity to gemcitabine or paclitaxel, and downregulated the expression *ABCB1* gene encoding for the integral membrane-associated protein, named Multidrug Resistance Protein 1, which is involved in extra- and intra-cellular membrane transport [26]. Finally, it has recently demonstrated that hsa-miR661-3p microRNA overexpression is able to decrease the growth rate in NSCLC through the downregulation of UbcH10 expression [23].

### 3.3. Breast Cancer

The UbcH10 protein was initially included in a high sensitivity and specific multimarker assay for the detection of circulating tumor cells (CTCs) in breast cancer patients [27]. Since then, there have been important progresses in breast cancer CTCs analysis, but in recent times the attention on UbcH10 involvement has declined [48,49]. Some studies based on tissue micro-arrays immunohistochemistry and human breast carcinoma-derived cell lines, have shown that high UbcH10 expression levels are associated with ductal histotype. These studies also showed ErbB2 immunoreactivity and high levels of Ki-67 staining in tumor tissues. In cell lines models, it has been shown that the specific small interfering RNAs (siRNAs)-mediated blocking of ErbB2 is able to downregulate the UbcH10 expression and significantly decreased the breast carcinoma cell growth [28]. The analysis of UbcH10 mRNA and protein content, analyzed in five breast cancer cell lines, confirmed that UbcH10 expression is significantly higher in comparison with normal mammary epithelial cells. Furthermore, the UbcH10 downregulation decreased the proliferation rate both in MCF-7 and in the epirubicin/docetaxel resistant cell line MCF-7/EPB/TXT cells, and in this last, also increased the epirubicin and docetaxel-induced apoptosis [29].

### 3.4. Thyroid Cancer

In thyroid cancer, it has been found that the UbcH10 protein is abundantly expressed in many thyroid carcinoma-derived cell lines, such as TPC-1, WRO, NPA, ARO, FRO, NIM 1, B-CPAP, FB-1, Kat-4 and Kat-18, whereas the protein levels are barely detectable in normal thyroid cells [30]. The expression of UbcH10 has been also analyzed in normal and neoplastic thyroid tissues by immunohistochemistry, revealing that normal thyroid, nodular goiter, and Hashimoto’s thyroiditis (HT) were almost always completely negative for UbcH10 expression. A weak staining was detectable in follicular adenomas and higher levels of UbcH10 were recorded in papillary (median value 2.2% of positive cells; range 0.9%–4.1%), follicular (median value 2.8% of positive cells; range 1%–6.1%) and poorly differentiated (median value 10.4% of positive cells; range 8%–14.9%) carcinomas. Mainly, the immunoreactivity signal has been always easily detectable in the nuclei of scattered neoplastic cells. Western blot analysis, performed on surgically removed thyroid tumors, confirmed the immunohistochemical data and RT-PCR analysis results [30]. Spectral karyotyping (SKY), G-banding and comparative genomic hybridization carried out in two novel anaplastic thyroid carcinoma (ATC) cell lines and in six frequently used ATC cell lines, detected a frequent gain of 20q, including the UBCH10 gene locus in 20q13.12 [31]. Interestingly, the amplification of the long arm of chromosome 20 has been frequently observed in many types of cancer in which an overexpression of UbcH10 is reported [50]. Finally, the analysis of UbcH10 in thyroid fine-needle aspiration (FNA) by quantitative RT-PCR is more useful than immunohistochemistry to increase the detection of malignancy in thyroid [32].

### 3.5. Gastroenteric Tumors

Immunohistochemical data obtained in squamous cells carcinoma of esophagus confirmed the general prognostic impact of UbcH10 evaluation in cancers. UbcH10 protein was detected in cancerous lesions and some dysplastic lesion, surrounding cancerous tissue, but not in normal tissue. Moreover, in patients with esophageal squamous cell carcinoma a significant association between high levels of UbcH10 protein and poor prognosis has been demonstrated [40].

High UbcH10 protein levels have also seen in gastric cancer tissues by immunohistochemistry in many cases and again, in adjacent normal tissues this protein was undetectable. Furthermore, studies on human gastric carcinoma cell lines such as SGC-7901, AGS, NCI-N87, HS 746T, MKN-45, KATO III, NCI-SNU-1, SNU-5, and SNU-16 demonstrated that siRNA-mediated knockdown of UbcH10 expression in gastric cancer cells reduced proliferation rate, increased cisplatin-induced apoptosis and decreased serum-induced ERK, Akt/PKB and p38 phosphorylation [41].

In colon cancer, all performed studies confirmed the importance of overexpression of UbcH10 in the process of tumorigenesis. In fact, the overexpression of UbcH10 in the DLD-1 colon cancer cell line led to a significant acceleration of cellular proliferation reducing the doubling times. Consistently, the UbcH10 knockdown, obtained by using specific siRNA, largely reduced cellular proliferation. Immunohistochemical tissue array analysis indicated that UbcH10 was significantly higher in colon cancer tissue compared to normal colon epithelia [33]. By using a short hairpin RNA expression cassette, containing a UbcH10 RNAi, it has been obtained the gene silencing in two colon cancer cell lines and, in vivo, in a nude mouse xenograft. In this work, cell growth was markedly suppressed, arresting in the G2-M phase. Moreover, the inhibition of tumour growth in the nude mice xenograft model has been obtained showing a therapeutic potential for targeting UbcH10 in colon cancer [34]. Other similar studies, in vitro and in vivo, employed the DDL-1 cells line and xenografts into nude mice and evaluated effects on proliferation by colony formation, growth curve, soft agar assay, and growth of xenograft implantation. The effect of blocking proteasome pathway by using N-acetyl-Leu-Leu-Norleu-al (ALLN) was also examined. Interestingly, high expression of UbcH10 drives resistance to ALLN-induced cell death, while cells deficient in UbcH10 are susceptible to ALLN-induced cell death. The depletion of UbcH10 hindered tumorigenesis, both in vitro and in vivo [35]. In a series of normal colon and colon carcinoma samples, collected from surgical specimens, UbcH10 mRNA and protein expression levels have been evaluated. The overexpression of UbcH10 mRNA and protein has been observed in the vast majority of patients analyzed. Moreover, the UbcH10 suppression analyzed in vitro study in Caco-2 and DLD-1 cell lines, decreases growth rate and sensitized them to pharmacological treatments with irinotecan, its active metabolite SN-38 and cetuximab [36]. Analysing the expression of UbcH10 protein in a series of colorectal cancer samples, matched with the corresponding normal tissue, obtained from elderly patients, it has been observed that the overexpression of UbcH10 is attenuated in relation to the patient’s age at surgery. Higher levels of UbcH10 overexpression were concentrated around 60 years of age, whereas it tended to lower in the groups of 70 and 80 years; in the same work, it has been also found that UbcH10 protein overexpression is related to the lymph node spread, as evaluated by N stage. Since the expression of UbcH10 predicts response to cetuximab, regardless of *ras* status, it would be worthy testing CRC patients also for UbcH10, prior to start treatments, and consider silencing UbcH10 as coadjuvant [37].

### 3.6. Gynecologic Tumors

In ovarian carcinomas, immunohistochemical studies demonstrate that UbcH10 expression significantly correlates with the tumor grade and the undifferentiated histotype. A clear correlation between UbcH10 overexpression and reduced survival in patients with ovarian carcinoma has been described and the indication of UbcH10 as a valid prognostic marker in this neoplastic disease has brought forth. The SKOV-3 cell line of ovary carcinoma has been treated with siRNA duplexes targeting the UbcH10 mRNA. After transfection, it was observed an efficient knock down of the UbcH10 protein levels. The cell growth analysis, in the presence or absence of siRNA duplexes, revealed that blocking UbcH10 protein synthesis, significantly inhibits ovarian carcinoma cell growth [38]. The UbcH10 expression has been studied in endometrial lesions by immunohistochemistry in curettage material. A statistically significant difference was found between a carcinoma group and other groups of benign lesions, such as proliferative endometrium, disordered proliferative endometrium, and non-atypical hyperplasia. Only differences with complex atypical hyperplasia group did not reach a statistically difference. Data collected in this tissue micro array-based studies, also in these cases, encourage to use UbcH10 as a diagnostic marker in histopathology [42].

### 3.7. Pancreas and Liver

Ductal adenocarcinoma of pancreas (PDA) is another solid neoplasm in which UbcH10 plays a pivotal role in tumorigenesis and clinical evolution of disease. Immunohistochemistry and RT-PCR showed that high expression of UbcH10 was significantly associated with poor overall survival in PDA patients. High expression of UbcH10 was also significantly correlated with the clinical stage, the degree of histological differentiation, and the presence of lymph node metastasis [39]. Hepatocellular carcinoma (HCC) has been studied for UbcH10 expression both in cancer and normal tissues. Several studies have been carried out in normal liver-derived cell line LO2 and cancer derived cell lines BEL-7402, Hep3B, HepG2, and SMMC-7721. Immunohistochemistry analyses identified stronger UbcH10 expression in hepatocellular carcinoma tissues compared to the adjacent tissues and normal liver tissue. Normal liver cell line showed significantly lower UbcH10 mRNA expression levels compared to the liver cancer derived cell lines. Moreover, UbcH10 mRNA expression levels were significantly higher in hepatocellular carcinoma tissues compared to the surrounding non-tumor tissues. Clinicopathological evaluation suggested that UbcH10 expression is associated with tumor invasion of the portal vein, tumor size, TNM staging, and tumor differentiation [51].

### 3.8. Other Neoplasms

Immunohistochemical studies in bladder cancer, allowed to observe UbcH10 positivity in 62% of bladder urothelial carcinoma cases treated with radical cystectomy. Instead, UbcH10 was undetectable in all non-neoplastic urothelium examined. UbcH10 positivity is significantly associated with higher tumor stage and presence of lymphovascular invasion. In addition, positivity has been also significantly associated with shorter cancer-specific survival after cystectomy [43].

In regard to studies of UbcH10 expression in bone osteosarcoma, the UbcH10 knockdown has been obtained in osteosarcoma derived U2OS and SaOS2 cell lines by using lentivirus-mediated RNA interference. The osteosarcoma cells that underwent to UbcH10 knockdown exhibited impaired invasion and migration capabilities. The downregulation of UbcH10 also suppressed osteosarcoma cell proliferation and colony formation ability decreasing Ki-67 expression. Furthermore, knockdown of UbcH10 led to decreased levels of extracellular matrix metalloproteinases [44].

RT-PCR and tissue microarray immunohistochemistry-based studies have been performed in a group of various type lymphomas. Cell lines and tissue samples both from Hodgkin’s (HL) l and of non-Hodgkin’s lymphoma (NHL) were assayed for UbcH10 expression at transcriptional and translational levels. UbcH10 expression has been found to be relatively low in indolent tumors and higher in a variety of aggressive lymphomas and HL- and NHL-derived cell lines. The highest levels were found in Burkitt’s lymphoma. UbcH10 expression plays a relevant role in lymphoid cell proliferation, since blocking its synthesis by RNA interference, inhibits cell growth [45]. Multiple myeloma is a pathology in which proteasome inhibitors have an important efficacy in the treatment. Recently it has been observed that UbcH10 is highly expressed in Bortezomib (BTZ)-resistant myeloma cell lines U-266/BTZ, NCI-H929/BTZ and RPMI-8226/BTZ evaluated in a gradient of BTZ increasing concentrations. In these cell lines it has been observed that during the development of BTZ resistance, the hsa-miR-631 levels are decreased and coherently the expression of the target gene UbcH10 increased [46].

## 4. Discussion

**Figure 1:**
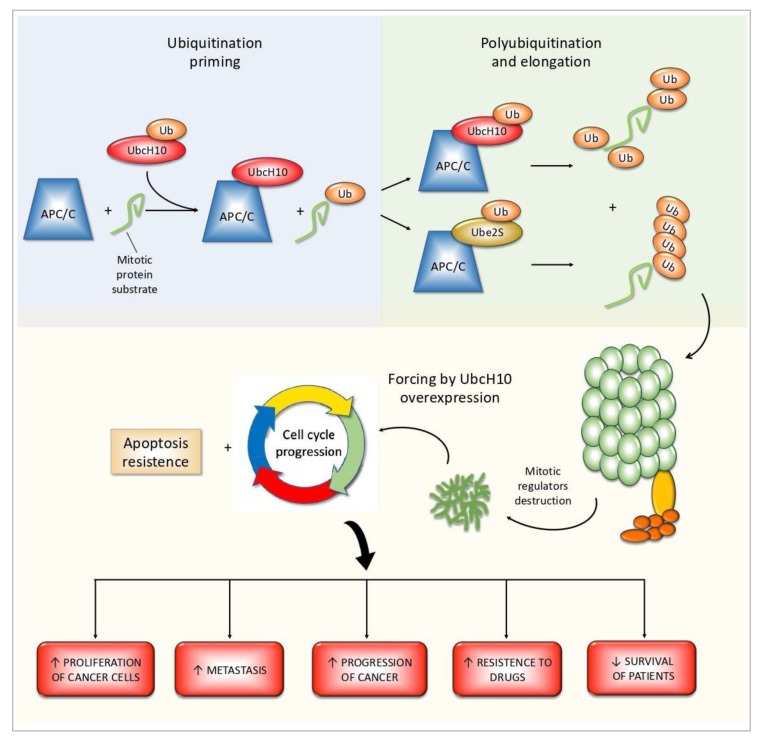
UbcH10 is together with Ube2S, the ubiquitin-conjugating enzymes (E2) partner of anaphase-promoting complex/cyclosome (APC/C) ubiquitin ligases (E3) complex. UbcH10 is devoted to ubiquitination priming and multi ubiquitination, while Ube2S presides to further ubiquitin chain elongation. UbcH10 overexpression has been detected in samples of diverse solid tumors or hematologic cancers as well as in experimental models. Experimental silencing of *UBE2C* mRNA levels allowed to find a link between its overexpression and worsening of malignant characteristics.

Taking into account the review of studies presented above, it appears extremely clear how the UbcH10 mitotic ubiquitin conjugating enzyme is a crucial common factor in the carcinogenesis process in many tumors (Figure 1).

Increased levels in mRNA and protein expression are well documented in most studies. As it has been shown in the diverse studies where the modulation of its expression is obtained, UbcH10 plays an essential role in the proliferation of cancer cells and probably in the progression of malignancies. In general, the immunohistochemical proofs about protein expression on tumor tissue samples correlates with the proliferative index and the degree of the neoplasms. Moreover, UbcH10 overexpression has been associated in transgenic mouse models, with distortions in cell division mechanics, such as supernumerary centrioles, lagging chromosomes, and aneuploidy [52]. The mechanisms by which UbcH10 is overexpressed in all various types of cancer are not fully understood. The hypothesis of the amplification of 20q chromosomal region, containing the *UBE2C* locus, is roughly obvious but is confirmed in studies carried out in colorectal and gastric cancer from microarray datasets and cancer cell lines. The gain of copy number on 20q almost always resulted in chromosomal instability [53,54]. Consistent with these findings, *UBE2C* has been included in the CIN25 chromosomal instability signature during a study employing several YAP-driven murine cell lines of cholangiocarcinoma [55]. Given this strong association between the gene amplification, or in any case the overexpression, of UbcH10 and the genomic instability observed in many types of cancer, it is reasonable that the positive selection of the UbcH10 hyper-expressing clones is a crucial event in the process of malignant transformation. It is probably one of the genes that promotes and supports heterogeneity in cancer cell populations with which poor prognosis and drug resistance are associated. The occurrence of other mechanisms, such as the control of gene expression based on epigenetics patterns, remains only a hypothetical and unexplored possibility. Many authors thought that the assessment of UbcH10 expression could be a useful diagnostic, prognostic and therapy-orienting tool. In fact, the immunohistochemical detection well characterizes only the neoplastic tissues, but not the corresponding normal tissues. Parallel data, coming from studies on mRNA levels, retraced the same expression profiles for neoplastic tissues. These results have been often correlated with clinical parameters, such as patient survival and disease staging. Regarding the possible therapeutic significance, the expression of UbcH10 has been shown to be a possible factor of resistance to therapy with certain drugs, including proteasome inhibitors. Initially, proteasome inhibitors were developed to prevent cancer-related cachexia and the first to be approved was bortezomib in 2003. At therapeutic doses, bortezomib inhibits approximately 30% of proteasome-mediated protein degradation [56], which is sufficient to induce tumor cell apoptosis without producing toxicity in noncancer cells. As aforementioned, recently, Xi and colleagues demonstrated, for the first time, that UbcH10 is highly expressed in three bortezomib (BTZ)-resistant multiple myeloma cell lines, which is attributed to the inactivation of post-transcriptional control. In particular, data revealed that during the development of bortezomib resistance in cells, the hsa-miR-631 levels were decreased, resulting in an increase of *UBE2C* gene expression. Furthermore, data showed that the multiple drug-resistant protein MDR1 exhibited a positive correlation with UbcH10 levels and researchers hypothesized the presence of a miR-631/UbcH10/MDR1 pathway during the development of BTZ resistance in multiple myeloma. However, in this study, it is not shown that the increased MDR1 protein stability is a direct effect of altered ubiquitination rate, eventually due to the UbcH10 overexpression and the actual mechanism of this association should be elucidated [46]. The mechanism by which proteasome inhibitors lead to cell death affects several pathways involved in cancer. One supposed cytotoxicity mechanism is represented by the inhibition of the pro-survival NF-κB pathway for several cell types, in particular for hematopoietic lineages. The endogenous NF-κB protein inhibitor IκBα is degraded by the proteasome and its degradation is necessary for the p50/p65 NF-κB transcription factors to become active and translocate to the nucleus [57]. All proteasome inhibitors primarily target the chymotrypsin-like, β5 subunit or, if used at higher concentrations, also block the trypsin-like (β2) and caspase-like (β1) subunits as well. More effective tools acting on this system are expected to increase efficacy in cancer therapy. There must be more specific UPS inhibitors targeting the core of ubiquitination’s specificity that reside in E2s or E3s components of the UPS system. A recent promising attempts have been made recently to target E1 enzymes [58], but the turning point could come from the studies about conjugating enzymes an ligases [59,60,61,62,63], hoping to get a specific inhibitor for UbcH10 to be placed alongside the specific siRNA or microRNA (miRNA) used so far. So far, only one study has been carried out to search for specific UbcH10 inhibitors but has stopped in the selection from ligand libraries identifying two candidate compounds that are waiting to be tested in vitro [64]. We would expect to emulate the results obtained with inhibitors of other E2 enzymes, such as in the case of small molecule inhibitor SMI # 9 for Rad6 in triple negative breast cancer. This molecule, beyond the problems faced in conveying the drug, has been shown to induce cytotoxicity in breast cancer cells but spares normal breast cells and demonstrated efficacy in combination with cisplatin [65]. In our opinion, the data accumulated in recent years concerning UbcH10 are solid and promising, and should not be forgotten. They show that UbcH10 has the potential for playing a pivotal role both as cancer marker and pharmacological target. By the way, the features of a so specific molecular marker must be exploited, integrating its detection with the rapidly developing techniques for the study of CTCs and circulating tumor nucleic acids; that can be combined with minimal invasiveness of liquid biopsy. The UbcH10 overexpression could be investigated directly on CTCs by immunocytochemical approaches, as demonstrated for other biomarkers [66,67,68,69], or by searching for its transcript in cell free circulating RNA in biological fluids. As it has been shown by recent, encouraging data from a study carried out on the urine of subjects with breast cancer [70], the analysis of *UBE2C* transcript levels could become a sensitive marker that can be exploited in diagnostics. More studies should be designed and performed in the near future to test the real power of what could be an exceptional tool for diagnostics and clinical practice.

## Figures and Tables

**Table 1:** List of significative papers about the role of UbcH10 in various cancers.

Tumor/Organ	Methods	Syntesis Results	References
**Intracranial meningioma**	Four primary meningioma cells cultures and Immunohistochemistry on tissue samples.	UbcH10 played an important role in the proliferation, apoptosis, and progression of human meningioma cells. RIZ1 regulated UbcH10 in a c-Myc dependent manner.	[18]
	Immunohistochemistry.	Nuclear and cytoplasm immunopositivity is observed in advanced stages; low immunoreactivity in meningiomas with low histological grade. UbcH10 immunoreactivity significantly overexpressed in meningioma patients with recurrence (*p* < 0.001).	[19]
**Glioma/Brain**	Cell culture of astrocytic tumors cells. Immunohistochemistry on tissue samples.	Elevated expression levels of UbcH10 messenger RNA (mRNA) in high- versus low-grade astrocytomas or normal controls. Immunohistochemistry shows increased UbcH10 in high-grade astrocytomas versus low-grade tumors or normal controls.	[20]
	Immunohistochemistry on tissue samples.	No UbcH10 detection in normal and gliotic brain. Correlation between UbcH10 expression and histological grade in astrocytic tumors.	[21]
	Cell culture of U251 human glioblastoma cells and small interfering RNA (siRNA) transfection.	RNA interference targeting UbcH10 induces growth inhibition, apoptosis, and cell cycle arrest of U251 cells.	[22]
**Carcinoma/Lung**	NSCLC tumors and cell lines A549 and SK-MES-1.	Impairment of NSCLC cells growth through the modulation of UbcH10 expression after hsa-miR661-3p restoration.	[23]
	Immunohistochemistry	UbcH10 positivity in lung adenocarcinoma, squamous cell carcinoma, large cell, and small cell carcinoma. Progressive increase of UbcH10 levels associated to decrease of tumor differentiation. Statistically significant difference in UbcH10 positivity between grade I/III of lung adenocarcinoma and squamous cell carcinoma.	[24]
	Quantitative RT-PCR and tissue microarray immunohistochemistry.	UbcH10 expression significantly higher in squamous cell and large cell carcinomas compared to adenocarcinomas. Opposite correlations with mutational status of p53 and EGFR.	[25]
	Immunohistochemistry Cell culture and transfection of siRNA.	UbcH10 overexpression in poorly differentiated NSCLC compared to well-differentiated ones. Higher levels in squamous cell carcinoma than in adenocarcinoma. Higher UbcH10 expression associated with shorter postoperative survival in NSCLC. Inhibition of cell proliferation and Increase of chemosensitivity after UbcH10 suppression in SK-MES-1 cells. Concomitant decrease of MDR1 gene expression.	[26]
**Carcinoma/Breast**	Array of mRNA markers expressed by circulating tumor cells (CTCs).	mRNA multimarker panel detected in CTCs from breast cancer patients included UbcH10.	[27]
	Tissue micro-arrays Immunohistochemistry MB231; MDA468; MDA436; MCF7; T47D and ZR 75-1 human breast carcinoma cell lines.	Association between high UbcH10 levels and ductal histotype. ErbB2 positivity and high Ki-67 staining. siRNA mediated UbcH10 downregulation inhibits breast carcinoma cell growth.	[28]
	Immunohistochemistry on cancer tissues. Molecular analysis in breast cancer cell lines.	Increased expression levels of UbcH10 in cancer, in comparison with adjacent tissues. UbcH10 mRNA and protein levels analysis in breast cancer cell lines are significantly higher compared to normal mammary epithelial cells. The knockdown of UbcH10 inhibits proliferation of MCF-7 and MCF-7/EPB/TXT cells increasing sensitivity to chemotherapy drugs.	[29]
**Carcinoma/Thyroid**	Immunohistochemistry on thyroid cancer tissues and normal thyroid tissues. RT-PCR and western blot analyses in human thyroid carcinoma cell lines.	Normal thyroid, nodular goiter, and HT almost always negative for UbcH10 expression. Weak staining in follicular adenomas; higher levels in papillary, follicular and poorly differentiated thyroid carcinomas. Abundancy of UbcH10 protein in diverse thyroid carcinoma cell lines, but barely detectable in normal thyroid cells. Western blot analysis, confirmed the immunohistochemical and RT-PCR data.	[30]
	Spectral karyotyping (SKY) and G-banding in two novel ATC lines and six frequently used ATC lines. Comparative genomic hybridization.	Frequent gain of 20q, including the *UBE2C* locus in 20q13.12.	[31]
	Immunohistochemistry and RT-PCR on thyroid FNA samples.	Quantitative RT-PCR is more useful than immunohistochemistry to evaluate UbcH10as marker of malignancy in thyroid FNAs.	[32]
**Carcinoma/Colon**	Cell-line based assay and tissue array analyses.	UbcH10 overexpression led to a significant acceleration of cellular growth in colon cancer cells. Knockdown of UbcH10 largely reduced cellular proliferation. Immunohistochemical analysis indicates that UbcH10 was significantly higher in colon cancer tissue compared to normal colon epithelia.	[33]
	Silencing *UBE2C* gene by RNAi in colorectal cancer cell growth in vitro and in vivo in a nude mouse xenografts model.	Suppression of colorectal cancer cells growth, with arrest in the G2-M phase, upon *UBE2C* gene silencing. The downregulation of *UBE2C* gene in vivo inhibited tumor growth in a nude mouse.	[34]
	DDL1 colorectal cancer cells line Xenograft into nude mice. Colony formation assay, growth curve, soft agar and xenograft assays. Blocking of proteasome pathway by ALLN.	High expression of UbcH10 drives resistance to ALLN-induced cell death, while cells deficient in UbcH10 are susceptible. The depletion of UbcH10 hindered tumorigenesis both in vitro and in vivo.	[35]
	UbcH10 mRNA and protein evaluation in colon carcinoma and normal samples from CRC patients. Growth assays and pharmacological treatments on Caco-2 and DLD-1 cell lines.	Overexpression of UbcH10 mRNA and protein observed in the vast majority of tumoral samples analyzed. UbcH10 suppression decreases Colorectal cancer cells growth rate and sensitizes them to pharmacological treatments with irinotecan, SN-38 and cetuximab.	[36]
	Analysis of protein expression in CRC samples and normal colonic tissue.	Levels of UbcH10 overexpression related to the age of patient at surgery and to the lymph node spread.	[37]
**Carcinoma/Ovary**	Immunohistochemistry on tumor tissues. Analysis on ovary carcinomas cell lines SKOV-3 also with siRNA targeting the *UBE2C* mRNA.	Cell growth inhibition after efficient knock down of the UbcH10 protein. Immunohistochemistry demonstrated that UbcH10 expression significantly correlates with the tumor grade. Clear correlation between UbcH10 overexpression and a reduced survival in ovarian carcinoma patients.	[38]
**Ductal adenocarcinoma/pancreas**	Real-time qRT-PCR Immunohistochemistry.	High levels of UbcH10 significantly associated with poor overall survival in PDA patients, with clinical stage, degree of histological differentiation, and lymph node metastasis.	[39]
**Esophagus/Squamous Cell Carcinoma**	Immunohistochemistry.	Detection of UbcH10 protein in cancerous lesions and some dysplastic lesions surrounding cancerous tissue, but not in normal tissue. association between high levels of UbcH10 protein expression in esophageal tissues and poor prognosis in patients with esophageal squamous cell carcinoma.	[40]
**Stomach/carcinoma**	Immunohistochemistry on cancer tissues. Gene silencing in human gastric carcinoma cell lines SGC-7901, AGS, NCI-N87, HS 746T, MKN-45, KATO III, NCI-SNU-1, SNU-5 and SNU-16.	Immunohistochemistry showed high levels of UbcH10 protein in most gastric cancer tissues but is unable to detect it in adjacent normal tissues. siRNA Knockdown of UbcH10 in gastric cancer cell lines (high expression of UbcH10) resulted in reduced proliferation, increased cisplatin-induced apoptosis, and reduced serum induced ERK, Akt, and p38 phosphorylation.	[41]
**Uterus/Carcinoma**	Immunohistochemistry on endometrial curettage biopsies of proliferative endometrium, disordered proliferative endometrium, complex atypical hyperplasia, nonatypical hyperplasia endometrioid adenocarcinoma.	A statistically significant difference was found only between the carcinoma group and the other groups, except the complex atypical hyperplasia group.	[42]
**Bladder/Cancer**	Immunohistochemistry on cancer tissues. Gene silencing in UM-UC-3 bladder cancer cell line.	SiRNA mediated suppression of UbcH10 in UM-UC-3 cells inhibited cell proliferation in vitro. Immunohistochemical UbcH10 positivity observed in 62% bladder urothelial carcinoma. Instead, UbcH10 negative in all non-neoplastic urothelium examined. UbcH10 positivity significantly associated with higher tumor stage and lymphovascular invasion. UbcH10 positivity significantly associated with shorter cancer-specific survival after cystectomy.	[43]
**Bone/Osteosarcoma**	UbcH10 knockdown in osteosarcoma U2OS and SaOS2 cell lines using lentivirus-mediated RNA interference.	UbcH10 knockdown in osteosarcoma cells exhibited impaired invasion and migration capacities. The downregulation of UbcH10 suppressed osteosarcoma cell proliferation and colony formation ability decreasing Ki-67 expression. Furthermore, UbcH10 knockdown led to decreased levels of matrix metalloproteases MMP-3 and MMP-9.	[44]
**Lymphoid cells/Lymphoma**	RT-PCR and tissue microarray immunohistochemistry to screening cell lines and tissue samples from HL and NHL for UbcH10 expression at transcriptional and translational levels.	Low UbcH10 expression in indolent tumors and higher expression in a variety of HL and NHL cell lines and in aggressive lymphomas. Highest expression in Burkitt’s lymphoma. UbcH10 plays a relevant role in lymphoid cell proliferation, since blocking of its synthesis by RNA interference inhibited cell growth.	[45]
**Multiple myeloma/Plasma cells**	Bortezomib (BTZ) -resistant myeloma cell lines U-266/BTZ, NCI-H929/BTZ and RPMI-8226/BTZ. Assessment of specific microRNAs (miRNAs) in both resistant and their parental cells. Luciferase reporter assay.	The development of BTZ resistance in U-266 cell lines is associated with decrease of hsa-miR-631 levels and increased expression of UbcH10. Positive correlation between MDR1 and UbcH10 due to reduced ubiquitination of MDR1. Overexpression of miR-631 enhanced both BTZ sensitivity and BTZ-induced apoptosis in resistant cells. Re-sensitization by miR-631 overexpression is blocked by exogenous UbcH10 not regulated by intracellular miR-631. Verification that hsa-miR-631 may inhibit translation by binding UbcH10-3’UTR.	[46]
**Hepatocellular carcinoma/Liver**	Immunohistochemistry in hepatocellular carcinoma tissue, the adjacent tissue and normal liver tissue. Study of UbcH10 mRNA expression using RT-PCR in normal liver cell line LO2, cancer cell lines BEL-7402, Hep3B, HepG2 and SMMC-7721.	Stronger UbcH10 expression in hepatocellular carcinoma tissues compared to adjacent tissues and normal liver tissues. Normal liver cell line showed relative lower UbcH10 mRNA levels compared to cancer cell lines. UbcH10 mRNA expression is significantly higher in hepatocellular carcinoma tissues compared to the corresponding non-tumor tissues. Clinicopathological evaluation suggests that UbcH10 expression is associated with tumor invasion of the portal vein, tumor size, TNM staging, and tumor differentiation.	[47]

Abbreviations. RIZ1: Retinoblastoma protein-interacting zinc finger protein1; c-Myc: proto-oncogene c-Myc, NSCLC: non-small cell lung carcinomas, EGFR: epidermal growth factor receptor, p53: tumor suppressor p53, HT: Hashimoto’s thyroiditis, HL: Hodgkin’s lymphoma; NHL: non-Hodgkin’s lymphoma; ATC: anaplastic thyroid carcinoma, FNA: fine-needle aspiration, CRC: colorectal cancer, PDA: Ductal adenocarcinoma of pancreas, BTZ: Bortezomib, TNM: TNM Classification of Malignant Tumors.

## Abbreviations

ABCB1	ATP binding cassette subfamily B member 1
Akt/PKB	AKT Serine/Threonine Kinase 1
ALLN	N-acetyl-Leu-Leu-Norleu-al
APC/C	Anaphase-promoting complex/cyclosome
ATC	Anaplastic thyroid carcinoma
BTZ	Bortezomib
Cdh1	APC coactivator Cdh1
CdK1	Cyclin-dependent kinase 1
CdK2	Cyclin dependent kinase 2
CDKs	Cyclin-depended kinases
c-Myc	Avian myelocytomatosis viral oncogene homolog
CTCs	Circulating tumor cells
E1s	Ubiquitin-activating enzymes
E2s	Ubiquitin-conjugating enzymes
E3s	Ubiquitin ligases
EGFR	Epidermal growth factor receptor
ErbB2	Erb-b2 receptor tyrosine kinase 2
ERK	Extracellular regulated MAP kinase
E2~Ub	E2 enzyme and ubiquitin conjugated by thioester bond
FNA	Fine-needle aspiration
HCC	Hepatocellular carcinoma
HL	Hodgkin’s lymphoma
hsa-miR-631	microRNA 631
hsa-miR661-3p	microRNA 661
NF-κB	Nuclear factor kappa B subunit
NHL	non-Hodgkin’s lymphoma
NSCLC	non-small cell lung carcinomas
p38	Mitogen-activated protein kinase p38
p53	Tumor suppressor p53
RIZ1	Retinoblastoma protein-interacting zinc finger protein1
RNAi	RNA interference
qRT-PCR	Quantitative real time polymerase chain reaction
siRNA	Small interfering RNA
SKY	Spectral karyotyping
UPS	Ubiquitin proteasome system
